# Radical Cystectomy with Ileal Conduit Urinary Diversion in a Patient with a Left Ventricular Assist Device

**DOI:** 10.1155/2015/484679

**Published:** 2015-07-28

**Authors:** Joseph J. Pariser, Adam B. Weiner, Gary D. Steinberg

**Affiliations:** Section of Urology, Department of Surgery, University of Chicago Pritzker School of Medicine, Chicago, IL 60637, USA

## Abstract

Left ventricular assist device (LVAD) is an option for the surgical management of severe heart failure, and radical cystectomy remains the standard of care for muscle-invasive bladder cancer. Given a complicated population in terms of comorbidities and management for patients with an LVAD, there is little experience with major urologic procedures, which require balancing the benefits of surgery with considerable perioperative risks. We report our experience performing the first radical cystectomy with ileal conduit in a patient with an LVAD and muscle-invasive bladder cancer.

## 1. Introduction

The LVAD can be utilized for severe heart failure as a “bridge” to heart transplantation or as “destination” therapy. It has been shown to improve survival and quality of life compared to medical therapy [1]. However, patient management is complicated because these patients require lifelong therapeutic anticoagulation and antiplatelet therapy. Undergoing elective noncardiac procedures in patients with an LVAD requires balancing the benefits of surgery with considerable perioperative risks. Radical cystectomy (RC) remains the mainstay of treatment for muscle-invasive bladder cancer. We present the first case of a RC with ileal conduit urinary diversion in a patient with an LVAD.

## 2. Case Presentation

An 81-year-old male with a history of chronic renal insufficiency, three myocardial infarctions, four coronary stents, and end-stage heart failure presented to an outside hospital in June 2013 for destination LVAD placement (HeartMate II, Thoratec). Prior to LVAD placement, he did not have hematuria. However, as an inpatient, he developed gross hematuria and ultimately underwent transurethral resection of bladder tumor. Pathology revealed muscle-invasive high-grade urothelial carcinoma (UC) with squamous differentiation. Metastatic workup included a CT chest, abdomen, and pelvis as well as a bone scan, which were both unremarkable.

After discharge, the patient's bladder cancer management was referred to our tertiary center, which is also an advanced heart failure referral center with considerable LVAD experience. By July 2013, he had recovered well from his LVAD placement with improved energy and exercise tolerance along with decreased dyspnea. Following a discussion of the risks and benefits, the decision was made for surgical resection with ileal conduit diversion. Neoadjuvant chemotherapy was considered but not pursued given the risks of thromboembolism and infection, preexisting renal insufficiency, and patient preference.

The patient was evaluated by anesthesia and cardiology preoperatively and cleared for surgery. He had previously been on aspirin 81 mg, but this was held for one week in preparation for surgery. The patient was bridged from warfarin to therapeutic enoxaparin (1 mg/kg twice daily) for 5 days with the last dose being 24 hours prior to surgery. In August 2013, the patient was preadmitted before surgery. Per protocol, he was given antimicrobial prophylaxis, alvimopan, and heparin 5000 u subcutaneous on the morning of surgery. Preoperative hemoglobin was 11.9 g/dL, and INR was 1.3.

After intubation, bilateral transversus abdominis blocks were performed for adjunctive pain control. Radical cystoprostatectomy, bilateral pelvic lymphadenectomy, and ileal conduit diversion were performed without complication. Intraoperatively, 3.6 L crystalloid, 250 mL 5% albumin, and 1 unit pRBC were administered. The patient had 265 mL of urine output and 600 mL of blood loss. A Jackson-Pratt drain and ureteral stents were left in place, and the patient was extubated. Total room time was 270 minutes, and time from incision to close was 133 minutes.

**Figure 1:**
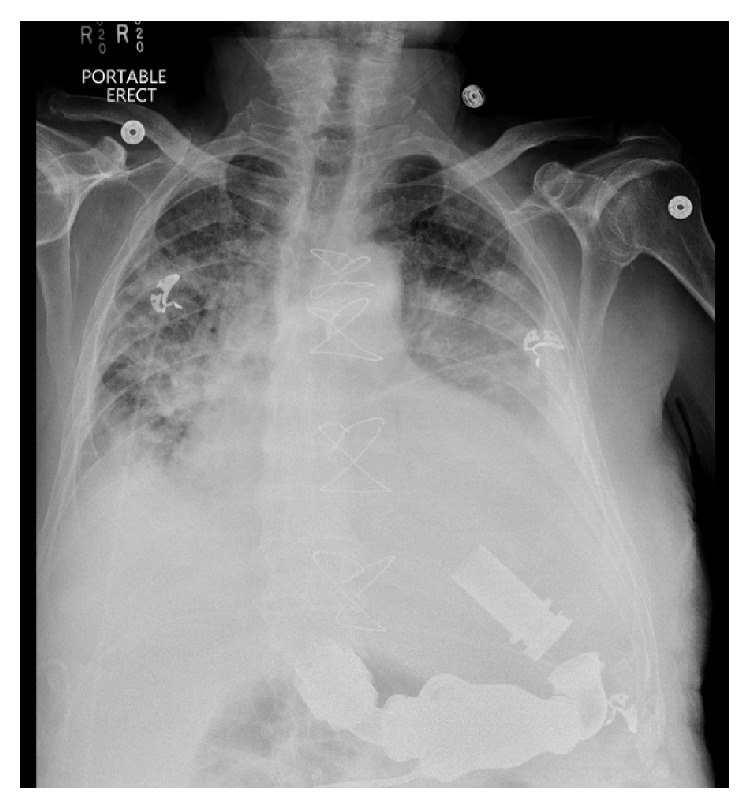
CXR showing severe cardiomegaly with diffuse interstitial and airspace opacities with effusions suggesting congestive heart failure. The patient's left ventricular assist device can be appreciated.

After surgery, the patient was admitted to the cardiothoracic intensive care unit (CICU) and given subcutaneous heparin every 8 hours. Postoperative investigations noted initially stable cardiopulmonary status (chest X-ray shown in Figure 1). Complete blood counts were drawn every 8–12 hours, and INR was checked daily. On postoperative day (POD) 1, he was transferred out of the CICU on aspirin 81 mg daily but no further anticoagulation given an INR of 2.1. By POD3, the patient's hemoglobin had decreased from 9.7 g/dL immediately after surgery to 7.5 g/dL. He was given 1 u pRBC and furosemide, but the patient became hypoxic. He was transferred back to the CICU and a bumetanide drip was initiated. He received another unit of blood once stabilized, and his hemoglobin increased to 10.7 g/dL.

**Figure 2:**
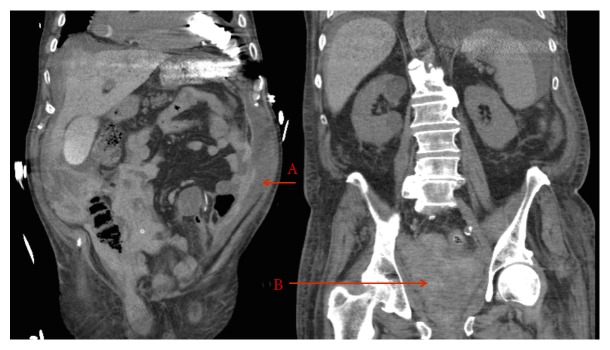
CT of abdomen and pelvis performed on POD11 demonstrating free fluid of mixed heterogeneity in the paracolic gutters (A) and pelvis (B) consistent with organizing hematoma.

The patient's diet was advanced on POD7 after passing flatus. From POD9 to POD11, there was a slow downtrend in hemoglobin. On POD11, a CT demonstrated free fluid of mixed heterogeneity in the paracolic gutters and pelvis, consistent with organizing hematoma (Figure 2). Over the next 72 hours, 4 units of pRBC and 4 units of FFP were given for anemia and an INR of 3.8. Afterwards, the patient required no further transfusions, suggesting a self-resolving bleed. He also underwent left thoracentesis for a pleural effusion with 1.8 L of straw-colored transudate removed. Despite maintained hemodynamics, the remainder of his hospitalization was prolonged due to pain (located near the surgical incision) and anticoagulation monitoring. The acute pain service was consulted on POD15 and assisted with inpatient pain management and the transition to a home analgesia regimen. Warfarin was initiated on POD13 at an INR of 1.5. On POD20, he was discharged with an INR of 2.0 and hemoglobin of 9.8 g/dL, stable over 6 days. Final pathology revealed T2aN0 high-grade UC with squamous differentiation, 26 negative nodes, and negative margins.

The patient's last follow-up was 18 months after cystectomy. Surveillance imaging showed no signs of recurrence. The patient continues to do well from a urologic and cardiac standpoint, including stable renal function (creatinine 1.4 mg/dL), satisfactory ostomy care, and appropriate activity tolerance.

## 3. Discussion

As the use of destination LVAD increases due to donor shortages and an aging population, there will likely be an increase in the number of patients with LVADs eligible for elective noncardiac surgery [2, 3]. However, given the considerable comorbidities of patients with an LVAD along with relatively shortened life expectancies, experience with noncardiac procedures remains limited. Previous urologic procedures in patients with an LVAD include partial nephrectomy [4], robotic prostatectomy [5], and nephroureterectomy (robotic [6] or laparoscopic [7]). Several insights can be garnered from our case.

Importantly, RC is the standard of care for muscle-invasive bladder cancer, but disease-specific and all-cause mortality declines following a delay in definitive surgical treatment greater than 12 weeks [8]. Therefore, a timely, multidisciplinary approach, as used for our patient, was necessary to maximize the benefit of surgery. Although the patient's age and comorbidities raised perioperative risks, the extensive experience from the anesthesia, CICU, and cardiothoracic surgery teams guided appropriate management. While a trimodal, bladder-sparing therapy (transurethral resection combined with radiotherapy and chemotherapy) can be considered in certain patients, it is associated with a significant risk of recurrence and need for salvage cystectomy. Additionally, radical cystectomy in the octogenarian has been shown to provide similar disease control and survival outcomes compared to younger patients in experienced centers [9].

In particular, management of hematologic complications required balancing needed anticoagulation with bleeding risks. Patients with an LVAD require full anticoagulation [10], but up to 58% experience major bleeding, commonly in the first three months following surgery [11]. Continuous-flow devices are associated with acquired von Willebrand syndrome [12, 13]. Additionally, RC carries a significant bleeding risk. In a series of 553 patients, Lowrance et al. reported that 38% of RC patients required transfusion with additional risk in older patients [14]. Our case demonstrates the need for constant vigilance towards hematologic and hemodynamic monitoring as well as the liberal use of transfusion when necessary to limit morbidity.

Variant histology, such as UC with squamous differentiation in this patient, predicts for invasive disease when discovered on transurethral biopsy [15] and is associated with a worse prognosis after RC than conventional UC [16]. At 18 months of follow-up, this patient has had an excellent outcome in terms of functional status and no recurrence of malignancy, suggesting a benefit from radical surgery.

For our patient with LVAD and invasive bladder cancer, RC with ileal conduit was feasible given an institutional experience in both realms. Such complicated procedures, with high perioperative morbidity, in patients with an LVAD should only be performed in tertiary centers with considerable expertise implanting and managing these devices. Our case highlights the perioperative morbidity of RC and the need for a multidisciplinary approach for these complicated patients in order to minimize complications related to thromboprophylaxis and fluid management.
